# Causal Relationship Between Cerebrospinal Fluid Metabolites and Intervertebral Disc Disease: A Bidirectional Mendelian Randomization Study

**DOI:** 10.3390/diagnostics15121526

**Published:** 2025-06-16

**Authors:** Jiheng Xiao, Tianyi Xia, Xianglong Zhou, Xin Xing, Yanbin Zhu, Yingze Zhang, Liming Xiong

**Affiliations:** 1 Department of Orthopaedics, Huazhong University of Science and Technology Tongji Medical College Union Hospital, Wuhan 430022, China; 2 Department of Orthopaedics, The Third Hospital of Hebei Medical University, Shijiazhuang 050051, China; 3 Key Laboratory of Orthopaedic Biomechanics of Hebei Province, Department of Orthopaedics, Shijiazhuang 050051, China; 4 Department of Clinical Medicine, Nankai University, Tianjin 300071, China

**Keywords:** causal inference, intervertebral disc degeneration, mendelian randomization, cerebrospinal fluid metabolites

## Abstract

**Background:** Intervertebral disc degeneration (IVDD) is caused by an imbalance between the catabolic and anabolic processes within intervertebral disc tissue. Several studies have suggested a potential association between cerebrospinal fluid metabolites (CFMs) and the development of IVDD. However, the existing evidence on the relationship between CFM and IVDD is limited and inconsistent. **Methods:** The data on 338 cerebrospinal fluid metabolites and intervertebral disc degeneration analyzed in this study were sourced from their respective genome-wide association studies (GWAS). MR analysis employed single nucleotide polymorphisms (SNPs) closely associated with disease as instrumental variables (IVs). The inverse variance weighted (IVW) method was employed as the primary statistical approach, complemented by MR-Egger, the Weighted median, Simple mode, and the Weighted mode for result validation. Comprehensive sensitivity analyses were performed to confirm the robustness of the results and assess for heterogeneity and horizontal pleiotropy. **Results:** Using the IVW method, this study revealed positive causal effects between 11 cerebrospinal fluid metabolites (CFMs) and intervertebral disc degeneration (IVDD), indicating that elevated levels of these 11 CFMs increase the risk of IVDD. Conversely, negative causal effects were identified for 6 CFMs, suggesting that higher levels of these CFMs have a protective effect against IVDD. Reverse MR analysis indicated 1 positive and 18 negative causal relationships between IVDD and CFMs. **Conclusions:** Our bidirectional Mendelian analysis provides compelling evidence of a causal relationship between CFMs and IVDD. These findings enhance our understanding of IVDD pathogenesis and highlight the potential for preventive therapies targeting CFMs. Further research is needed to clarify the mechanisms of these CFMs on IVDD.

## 1. Introduction

Intervertebral disc degeneration (IVDD) is a common, age-associated condition stemming from metabolic disturbances, characterized by the progressive depletion of proteoglycans and water in the nucleus pulposus, potentially culminating in disc rupture among vertebrae [1,2]. Degenerated discs are more susceptible to herniation, which can impinge on spinal nerves and cause symptoms such as low-back pain and sciatica [3]. IVDD, although sometimes asymptomatic, is often associated with disc herniation, low back pain, and lumbar radiculopathy, particularly affecting the L4-S1 nerve roots [4]. This underscores the significance of understanding the pathogenesis and risk factors involved in IVDD for effective management and treatment strategies. Emerging evidence indicates a potential involvement of metabolomics disturbances in the pathogenesis of IVDD [5,6].

Early studies have indicated an association between abnormal metabolites and the subsequent development of IVDD [7,8,9]. Among various metabolites, cerebrospinal fluid (CSF) is often used by researchers to explore the interaction with the nervous system because of its unique physical and chemical properties [10]. For instance, Dong et al. derived principal components from 308 CSF metabolites and presented evidence of the association between CSF metabolites and Alzheimer’s disease biomarkers [11]. Recently, Panyard et al. conducted a metabolome-wide association study (MWAS) on 338 CSF metabolites, performing metabolome-wide and genome-wide association analyses, identifying 16 genotype-metabolite associations, testing them against 27 neurologic and psychiatric phenotypes, and discovering 19 significant CSF metabolite–phenotype associations, further confirming a potential close link between CSF and neurologic disorders [12]. However, there is currently no research exploring the mutual association between CSF and IVDD, possibly due to challenges in obtaining CSF and conducting metabolic studies. There is no definitive conclusion at the genetic level regarding a causal relationship between cerebrospinal fluid metabolites and intervertebral disc degeneration, which has led to inadequate academic attention being paid to this issue.

**Figure 1:**
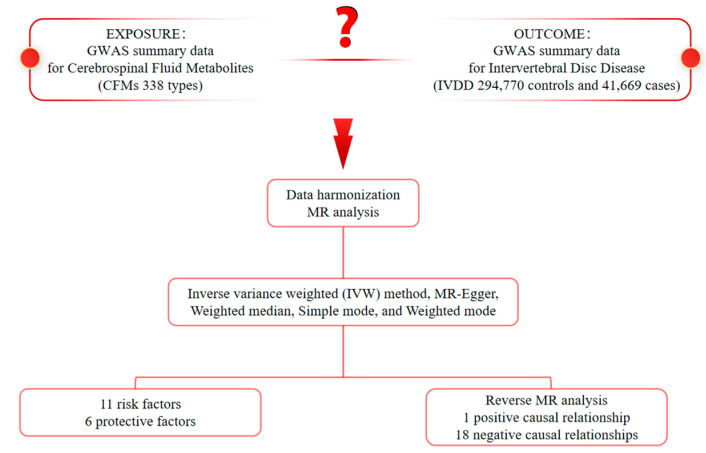
Diagram of the causal relationship between CFMs and IVDD.

Mendelian randomization (MR) is a crucial analytical method for inferring etiology in epidemiological studies [13]. It is grounded in Mendel’s law of independent assortment [14]. MR employs genetic variation as an instrumental variable to evaluate the impact of risk factors on outcomes [15]. By maintaining a plausible causal sequence, MR effectively mitigates biases associated with confounding and reverse causality, distinguishing it from other statistical approaches [16]. Hence, this study used a dual-sample Mendelian randomization approach to analyze cerebrospinal fluid metabolites and osteoporosis samples, aiming to elucidate their causal relationship. This could connect cerebrospinal fluid metabolites to IVDD pathogenesis, offering novel insights into understanding and treating IVDD (Figure 1).

## 2. Materials and Methods

### 2.1. Study Design

Forward and reverse Mendelian randomization analyses were used to investigate the bidirectional causal effects of 338 cerebrospinal fluid and intervertebral disc degeneration. Three important assumptions need to be made when analyzing Mendelian randomization: (1) the assumption of correlation, meaning the assumption that there is a solid correlation between the instrumental variable (IVs) and the exposure factor; (2) the assumption of independence, assuming that the instrumental variable is independent of confounders; and (3) the assumption of exclusivity, claiming that the instrumental variable can only have an effect on the outcome through the exposure factor [17]. Exposure refers to the potential risk factors explored in the study or variables that may affect the outcome, which is strongly correlated with the instrumental variables. The outcome is the health outcome or disease state that is being looked at in the study and is the endpoint variable that the exposure may affect. In the article, single nucleotide polymorphisms (SNPs) associated with cerebrospinal fluid metabolite levels were used as instrumental variables to test whether these SNPs affect the risk of disc degeneration by altering metabolite levels. With this approach, Mendelian randomization analyses can help determine a causal relationship between exposure (cerebrospinal-fluid metabolite levels) and outcome (intervertebral disc degeneration) rather than a simple correlation. In addition, all data used in the analysis came from publicly available and previously published datasets, so no ethical approval or informed consent was required.

### 2.2. Genome-Wide Association Study (GWAS) Data Sources for IVDD

The GWAS data used in this study were obtained from the Integrative Epidemiology Unit (IEU) Open GWAS project, managed by the Medical Research Council (MRC) Integrative Epidemiology Unit at the University of Bristol. This initiative integrates and analyzes GWAS datasets from various sources, including the UK Biobank, peer-reviewed studies, and the FinnGen biobank. Published GWAS studies had passed ethical review at the time of study and all data used were publicly available, anonymized, and de-identified. Ethical approval from an institutional review board was not required for our study, as it adhered to standard ethical guidelines.

Summary-level data for intervertebral disc degeneration (IVDD) were sourced from the FinnGen database R10 (https://r10.finngen.fi/pheno/M13_INTERVERTEB, accessed on 6 December 2024), comprising 41,669 cases and 294,770 controls. The disease phenotype for IVDD was defined based on the International Classification of Diseases (ICD) coding system, including the Tenth (ICD-10), Ninth (ICD-9), and Eighth (ICD-8) editions. FinnGen’s phenotype library categorizes diseases according to the ICD-10, ICD-9, and ICD-8 codes, ensuring the reliability of the data for subsequent analyses. For this study, IVDD data were classified using the following ICD definitions: ICD-10 M51 refers to intervertebral disc disease of the thoracic, thoracolumbar, and lumbosacral spine; ICD-9 722 encompasses intervertebral disc disorders; and ICD-8 725 defines intervertebral disc displacement or slippage.

### 2.3. CFMs GWAS Data Sources

We utilized the most comprehensive and extensive collection of CFMs-related GWAS Catalog data (https://www.ebi.ac.uk/gwas/studies/GCST90025999, https://www.ebi.ac.uk/gwas/studies/GCST90026336, accessed on 6 December 2024). The study by Panyard DJ et al. measured cerebrospinal fluid metabolites in 689 participants, totaling 338 CFMs (PubMed ID: 33437055) [12].

### 2.4. Selection of Instrumental Variables (IVs)

To ensure compliance with the association hypothesis of Mendelian randomization analysis, (1) SNPs with a strong correlation with exposure were selected using a *p* < 5 × 10^−8^ threshold; (2) the threshold parameters were set (R^2^ = 0.001, kb = 10,000) to avoid the effect of chain disequilibrium; then, SNPs with allele frequencies less than 0.01 were excluded, and duplicates and palindromic SNPs were removed. (3) In order to reduce the bias caused by weak instrumental variables, the F statistic was chosen to assess the strength of association between instrumental variables and inflammatory proteins, calculated as F = [R^2^(N − 1 − K)]/[K × (1 − R^2^)], where R^2^ = β^2^ × 2 × MAF × (1 − MAF), and N denotes the sample size. SNPs with an F value lower than 10 were excluded to minimize the bias caused by the weak instrument; (4) SNPs associated with confounders and disc degeneration outcomes were manually screened and removed [18].

### 2.5. Statistical Analysis

All analyses were conducted using R 4.3.2 software. To investigate the causal relationship between candidate functional metabolites (CFMs) and intervertebral disc degeneration (IVDD), we employed several Mendelian Randomization (MR) methods, including the inverse variance weighted (IVW) method, the MR-Egger method, the weighted median (WM), and weighted mode approaches [19]. Among these, the primary method was IVW analysis, which assumes no intercept term in the regression model and utilizes outcome variance to provide reliable estimates, particularly in the absence of directed pleiotropy of instrumental variables (IVs) [20]. Heterogeneity across the selected IVs was assessed using Cochran’s Q statistic, with corresponding *p*-values. If the null hypothesis was rejected, we used the random-effects IVW model. To identify and exclude potential horizontal pleiotropic outliers that could significantly affect the results, we applied both the MR-Egger and MR-PRESSO methods [21,22]. Additionally, leave-one-out and scatter plots demonstrated that outliers had minimal influence on the results, underscoring the robustness and consistency of the findings, as well as the absence of significant heterogeneity in the correlations.

## 3. Results

### 3.1. Exploration of the Causal Effect of CFM on IVDD

**Figure 2:**
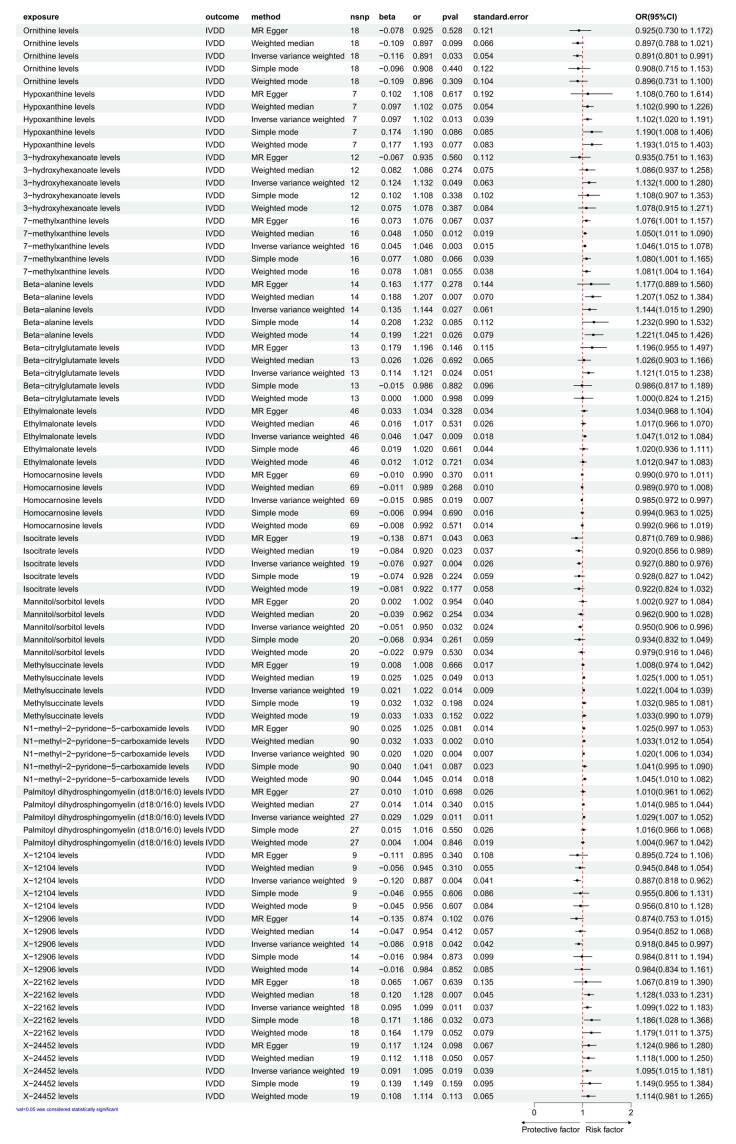
The forest map shows a causal relationship between CFMs and IVDD. IVW, inverse variance weighting; CI, confidence interval.

As illustrated in Figure 2, based on the selection criteria for SNPs and the *p* values denoting significance, we identified 17 CFMs with causal relationships with IVDD. These SNPs could serve as IVs in the MR analysis. We employed IVW as the primary analytical method. The IVW analysis detected 11 CFMs as risk factors for IVDD. The specific MR results are as follows: hypoxanthine levels (OR, 1.132; 95% CI, 1.000 to 1.280; nsnp = 7; β = 0.097; *p* = 0.013; SE = 0.039); 3-hydroxyhexanoate levels (OR, 1.102; 95% CI, 1.020 to 1.191; nsnp = 12; β = 0.124; *p* = 0.049; SE = 0.063); 7-methylxanthine levels (OR, 1.046; 95% CI, 1.015 to 1.078; nsnp = 16; β = 0.045; *p* = 0.003; SE = 0.015); beta-alanine levels (OR, 1.144; 95% CI, 1.015 to 1.290; nsnp = 14; β = 0.135; *p* = 0.027; SE = 0.061); beta-citrylglutamate levels (OR, 1.121; 95% CI, 1.015 to 1.238; nsnp = 13; β = 0.114; *p* = 0.024; SE = 0.051); ethylmalonate levels (OR, 1.047; 95% CI, 1.012 to 1.084; nsnp = 46; β = 0.046; *p* = 0.009; SE = 0.018); methylsuccinate levels (OR, 1.022; 95% CI, 1.004 to 1.039; nsnp = 19; β = 0.021; *p* = 0.014; SE = 0.009); N1-methyl-2-pyridone-5-carboxamide levels (OR, 1.020; 95% CI, 1.006 to 1.034; nsnp = 90; β = 0.020; *p* = 0.004; SE = 0.007); palmitoyl dihydrosphingomyelin (d18:0/16:0) levels (OR, 1.029; 95% CI, 1.007 to 1.052; nsnp = 27; β = 0.029; *p* = 0.011; SE = 0.011); X-22162 levels (OR, 1.099; 95% CI, 1.022 to 1.183; nsnp = 18; β = 0.095; *p* = 0.011; SE = 0.037); and X-24452 levels (OR, 1.095; 95% CI, 1.015 to 1.181; nsnp = 19; β = 0.091; *p* = 0.019; SE = 0.039). Additionally, the IVW analysis identified six CFMs that serve as protective factors against IVDD: ornithine levels (OR, 0.891; 95% CI, 0.801 to 0.991; nsnp = 18; β = −0.116; *p* = 0.033; SE = 0.054); homocarnosine levels (OR, 0.985; 95% CI, 0.972 to 0.997; nsnp = 69; β = −0.015; *p* = 0.019; SE = 0.007); isocitrate levels (OR, 0.927; 95% CI, 0.880 to 0.976; nsnp = 19; β = −0.076; *p* = 0.004; SE = 0.026); mannitol/sorbitol levels (OR, 0.950; 95% CI, 0.906 to 0.996; nsnp = 20; β = −0.051; *p* = 0.032; SE = 0.024); X-12104 levels (OR, 0.887; 95% CI, 0.818 to 0.962; nsnp = 9; β = −0.120; *p* = 0.004; SE = 0.041); and X-12906 levels (OR, 0.918; 95% CI, 0.845 to 0.997; nsnp = 14; β = −0.086; *p* = 0.042; SE = 0.042). Pleiotropy analysis (Table 1) showed that the MR-Egger test function results for the 17 CFMs with IVDD had *p* values greater than 0.05, indicating no significant effects. Data visualization in Supplementary Figure S1A–K indicates a positive slope (greater than 0) for the overall SNP effect of exposure factors such as hypoxanthine, 3-hydroxyhexanoate, 7-methylxanthine, beta-alanine, beta-citrylglutamate, ethylmalonate, methylsuccinate, N1-methyl-2-pyridone-5-carboxamide, palmitoyl dihydrosphingomyelin (d18:0/16:0), X-22162, and X-24452 levels, suggesting an increase in the SNP effect of the outcome variable IVDD. Conversely, exposure factors such as ornithine, homocarnosine, isocitrate, mannitol/sorbitol, X-12104, and X-12906 levels show a negative slope (less than 0), indicating a decrease in the SNP effect of IVDD (Figure S1L–Q). When the SNP effect of exposure is zero, the outcome variable is also zero, supporting that our study is not influenced by horizontal pleiotropy. For heterogeneity detection, IVW analysis of the 17 CFMs with IVDD showed *p* > 0.05, suggesting no heterogeneity among SNPs. The funnel plot (Figure S2) appears roughly symmetrical, indicating minimal bias due to heterogeneity in this study. In the heterogeneity Q test (Table 2), the IVW method for the 17 CFMs and IVDD showed *p* > 0.05, indicating no heterogeneity among SNPs. Leave-one-out analyses showed similar IVW results with all SNPs included, identifying no SNPs significantly influencing the causal estimates (Figure S3).

### 3.2. Exploration of the Causal Effect of IVDD on CFM

**Figure 3:**
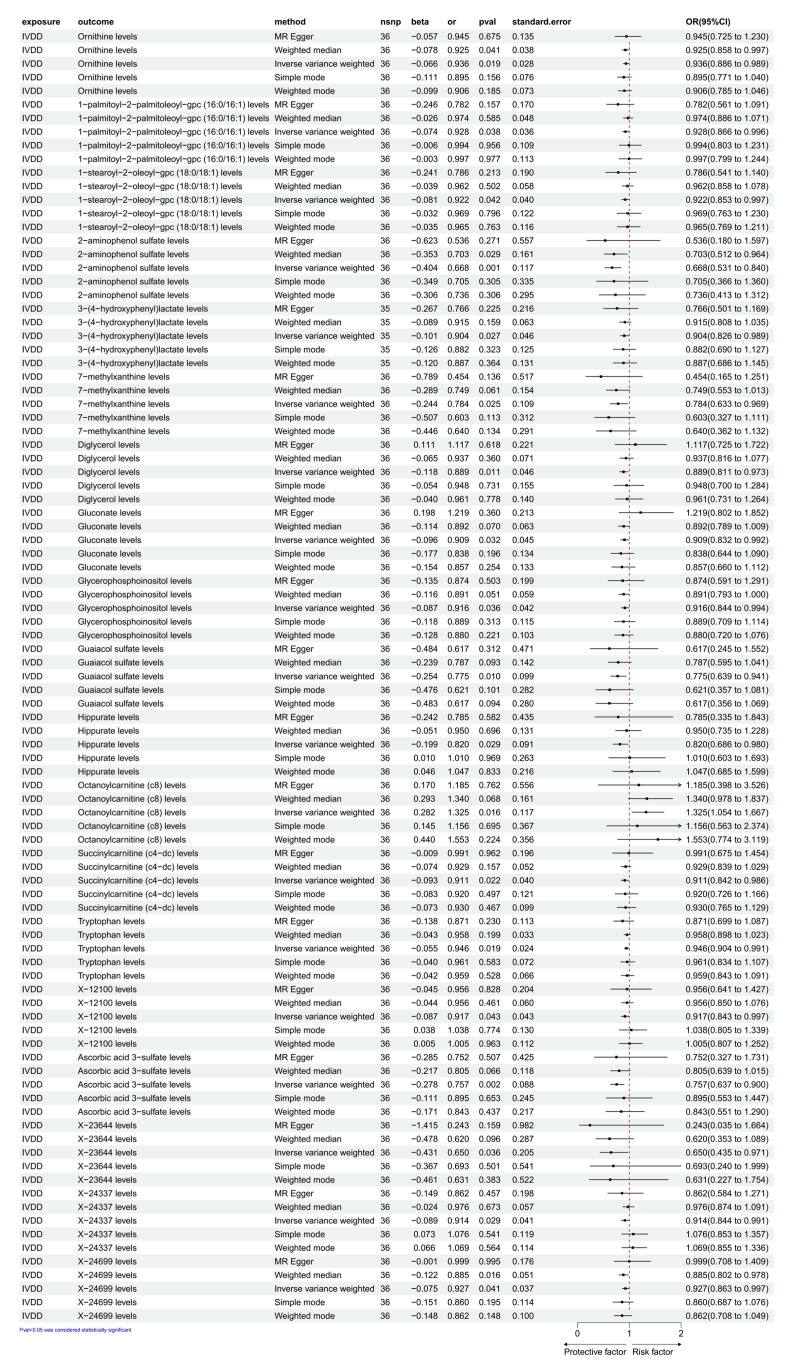
The forest map shows a causal relationship between IVDD and CFMs. IVW, inverse variance weighting; CI, confidence interval.

As shown in Figure 3, with IVDD as the exposure factor, a total of 36 SNPs were strongly correlated and used as instrumental variables (IVs) for subsequent analysis. Based on significant *p* values, we detected a causal relationship between IVDD and 19 types of CFM. According to the IVW analysis results, we found that IVDD can cause the production of only one cerebrospinal fluid metabolite. The specific MR analysis results are as follows: octanoylcarnitine (c8) levels (OR, 1.325; 95% CI, 1.054~1.667; nsnp = 36; β = 0.282; pval = 0.016; SE = 0.117). And then, we detected that IVDD might decrease the levels of 18 types of CFM: ornithine levels (OR, 0.936; 95% CI, 0.886~0.989; nsnp = 36; β = −0.066; pval = 0.019; SE = 0.028); 1-palmitoyl-2-palmitoleoyl-gpc (16:0/16:1) levels (OR, 0.928; 95% CI, 0.866~0.996; nsnp = 36; β = −0.074; pval = 0.038; SE = 0.036); 1-stearoyl-2-oleoyl-gpc (18:0/18:1) levels (OR, 0.922; 95% CI, 0.853~0.997; nsnp = 36; β = −0.081; pval = 0.042; SE = 0.040); 2-aminophenol sulfate levels (OR, 0.668; 95% CI, 0.531~0.840; nsnp = 36; β = −0.404; pval = 0.001; SE = 0.117); 3-(4-hydroxyphenyl) lactate levels (OR, 0.904; 95% CI, 0.826~0.989; nsnp = 36; β = −0.101; pval = 0.027; SE = 0.046); 7-methylxanthine levels (OR, 0.784; 95% CI, 0.633~0.969; nsnp = 36; β = −0.244; pval = 0.025; SE = 0.109); diglycerol levels (OR, 0.889; 95% CI, 0.811~0.973; nsnp = 36; β = −0.118; pval = 0.011; SE = 0.046); gluconate levels (OR, 0.909; 95% CI, 0.832~0.992; nsnp = 36; β = −0.096; pval = 0.032; SE = 0.045); glycerophosphoinositol levels (OR, 0.916; 95% CI, 0.844~0.994; nsnp = 36; β = −0.087; pval = 0.036; SE = 0.042); guaiacol sulfate levels (OR, 0.775; 95% CI, 0.639~0.941; nsnp = 36; β = −0.254; pval = 0.010; SE = 0.099); hippurate levels (OR, 0.820; 95% CI, 0.686~0.980; nsnp = 36; β = −0.199; pval = 0.029; SE = 0.091); succinyl carnitine (c4-dc) levels (OR, 0.911; 95% CI, 0.842~0.986; nsnp = 36; β = −0.093; pval = 0.022; SE = 0.040); tryptophan levels (OR, 0.946; 95% CI, 0.904~0.991; nsnp = 36; β = −0.055; pval = 0.019; SE = 0.024); X-12100 levels (OR, 0.917; 95% CI, 0.843~0.997; nsnp = 36; β = −0.087; pval = 0.043; SE = 0.043); ascorbic acid 3-sulfate levels (OR, 0.757; 95% CI, 0.637~0.900; nsnp = 36; β = −0.278; pval = 0.002; SE = 0.088); X-23644 levels (OR, 0.650; 95% CI, 0.435~0.971; nsnp = 36; β = −0.431; pval = 0.036; SE = 0.205); X-24337 levels (OR, 0.914; 95% CI, 0.844~0.991; nsnp = 36; β = −0.089; pval = 0.029; SE = 0.041); and X-24699 levels (OR, 0.927; 95% CI, 0.863~0.997; nsnp = 36; β = −0.075; pval = 0.041; SE = 0.037). In the pleiotropy analysis (Table 3), the MR-Egger test function results for IVDD and the 19 types of CFM suggested that the *p* value was greater than 0.05. Data-visualization analysis indicated that under the IVW method, the overall SNP effect of the exposure factor IVDD increased, and the SNP effect of the outcome variable octanoylcarnitine (c8) levels also increased (slope greater than 0) (Figure S4A). In contrast, the overall SNP effect of the exposure factor IVDD increased, while the SNP effects of the outcome variables decreased, including ornithine levels; 1-palmitoyl-2-palmitoleoyl-gpc (16:0/16:1) levels; 1-stearoyl-2-oleoyl-gpc (18:0/18:1) levels; 2-aminophenol sulfate levels; 3-(4-hydroxyphenyl) lactate levels; 7-methylxanthine levels; diglycerol levels; gluconate levels; glycerophosphoinositol levels; guaiacol sulfate levels; hippurate levels; succinyl carnitine (c4-dc) levels; tryptophan levels; X-12100 levels; ascorbic acid 3-sulfate levels; X-23644 levels; X-24337 levels; and X-24699 levels (slope less than 0) (Figure S4B–S). These results suggest that this study is not affected by horizontal pleiotropy. In heterogeneity testing, the IVW *p* > 0.05 for IVDD and 19 types of CFM, suggesting no heterogeneity among the SNPs. The funnel plot showed an approximately symmetrical shape, indicating a low possibility of result bias due to heterogeneity in this study (Figure S5). In the heterogeneity Q test (Table 4) and after sequentially excluding SNPs using the leave-one-out method, the IVW analysis results for IVDD and 19 types of CFM were similar to those when all of the SNPs were included. No SNP with a significant impact on causal association estimates was found, indicating the stability of the causal associations shown in the analysis results (Figure S6).

## 4. Discussion

IVDD is a leading cause of low back pain and a common degenerative disease [23]. While IVDD’s exact etiology remains unclear, multiple factors contribute, including genetics, mechanical load, inflammation, nutrition, and aging [24,25,26]. Early IVDD research emphasized mechanical stress, nutrient deficits in annulus fibrosus/cartilaginous endplate, and nucleus pulposus degeneration [27]. Recent IVDD research has increasingly focused on ECM alterations, impaired MSC differentiation, and inflammatory dysregulation [28]. For example, during IVDD, the increased release of inflammatory factors such as TNF-α, IL1-β, interleukin (IL)-6, IL-8, and prostaglandin E2 (PGE2) is stimulated by high catabolic metabolism and low synthetic metabolism of ECM [29,30,31]. Likewise, a decreased nutrient supply leads to an acidic shift in the IVD microenvironment, further exacerbating ECM degradation and pro-inflammatory cytokine release [32]. Hence, the pathologic process of IVDD often includes inflammatory reactions in the intervertebral disc and surrounding tissues. Furthermore, studies have shown that during cellular senescence, cells cease proliferation while persistently releasing various inflammatory factors (including homocarnosine) and matrix metalloproteinases, thereby exacerbating intervertebral disc degeneration [33]. Activated inflammatory cells secrete cytokines, maintaining the inflammatory microenvironment. These inflammatory mediators can penetrate the blood–brain barrier (BBB) or the cerebrospinal fluid–spinal cord barrier, inducing alterations in specific metabolites within the cerebrospinal fluid.

CSF, a crucial part of the central nervous system (CNS), possesses distinct physiological properties that can impact the development and advancement of neurovascular diseases. CSF biochemically reflects CNS status and, given its spinal proximity, may influence or mirror adjacent pathology. Studies have demonstrated significant shifts in CSF metabolic profiles in degenerative and inflammatory diseases. For instance, Horvatić et al. [34] applied a multi-omics approach in canine disc herniation and observed distinct metabolic changes in CSF, implicating energy metabolism and amino-acid dysregulation in disc pathology. Studies by Kosek et al. report elevated levels of IL-8 in the CSF of patients with knee osteoarthritis [35], suggesting that there may be a strong association between certain cerebrospinal fluid components and other inflammatory responses throughout the body. Furthermore, research indicates that alterations in CSF components can lead to a reduction in the extracellular matrix in soft tissues, triggering degenerative changes and inflammatory reactions. For example, elevated levels of the CSF metabolite homocysteine lead to the accumulation of toxic free radicals at the BBB, thereby activating matrix metalloproteinases (MMPs) [36,37]. MMPs critically degrade ECM, with increased activity releasing collagen fragments and proteoglycans into CSF, altering its composition [38].

This research marks the first exploration of bidirectional causality between 338 CFMs and IVDD, employing Mendelian randomization with a broad, publicly accessible genetic dataset. Based on the results of MR analysis, we found that 11 metabolites, including hypoxanthine levels, were risk factors for IVDD, and 6 metabolites, including ornithine levels, were protective factors, suggesting that patients with high indicators of above 11 metabolites should be alerted to the risk of subsequent IVDD. Notably, elevated levels of hypoxanthine, a purine metabolism intermediate, were causally linked with increased IVDD risk. This aligns with previous research indicating that hypoxanthine accumulation contributes to oxidative stress and mitochondrial dysfunction, both of which are detrimental to disc cell viability [39]. Conversely, ornithine, a key component in the urea-cycle and tissue-regeneration pathways, exhibited a protective effect against IVDD in both forward and reverse MR analysis. This is consistent with the recent literature emphasizing ornithine’s role in anti-inflammatory responses and collagen synthesis, potentially aiding in ECM repair and maintaining disc integrity [40]. Our reverse MR analysis revealed that IVDD could lead to increased levels of octanoylcarnitine (C8) and decreased levels of 18 CFMs, including gluconate and glycerophosphoinositol. These changes may represent downstream metabolic responses secondary to chronic inflammation, altered vascular perfusion, and ECM catabolism within degenerated discs. For example, a decline in gluconate—a compound with osmoprotective and antioxidant roles—may reflect the oxidative microenvironment prevalent in IVDD [41]. Importantly, the metabolites identified in this study intersect key biological pathways, including the urea cycle, fatty acid oxidation, purine metabolism, and mitochondrial energy regulation—all of which have been previously implicated in disc degeneration and neuroinflammatory responses [42].

Our findings also emphasize the potential bidirectional relationship between systemic metabolism and localized spinal pathology. Prior metabolomics reveals that disc-related metabolic changes actively drive, rather than merely reflect, degeneration progression [43]. Collectively, this is the first MR-based study to explore the causal interplay between CFMs and IVDD across both directions. These insights underscore the need to further investigate CSF metabolite profiles, not only as biomarkers for early IVDD detection but also as potential therapeutic targets for halting or reversing disc degeneration. Therapeutic strategies aimed at modulating CFM levels, such as enhancing ornithine bioavailability or mitigating purine-related oxidative stress, could complement existing mechanical and surgical interventions. In light of these findings, future work should integrate longitudinal metabolomics, multi-tissue transcriptomics, and functional validation models to unravel the precise mechanistic links between CSF metabolites and intervertebral disc health.

## 5. Limitation

Despite the strengths of this bidirectional Mendelian randomization (MR) analysis, several limitations should be acknowledged. First, while our analysis identified several cerebrospinal fluid metabolites (CFMs) potentially causally associated with intervertebral disc degeneration (IVDD), the underlying biological mechanisms remain largely undefined. Their roles in disc-matrix homeostasis, inflammation, or cellular metabolism have not been fully elucidated and warrant further mechanistic investigation in vitro and in vivo. Second, a fundamental assumption of MR is that the selected genetic instruments are valid—i.e., strongly associated with the exposure, independent of confounders, and exerting effects on the outcome exclusively through exposure. While we employed stringent quality control and sensitivity analyses to mitigate bias, the possibility of horizontal pleiotropy and residual confounding cannot be entirely excluded. Third, MR estimates represent the lifelong effect of genetically proxied exposure. This time-invariant assumption may not adequately capture the dynamic nature of metabolite levels, which can fluctuate due to metabolic status, disease stage, or environmental influences. This physiological variability could obscure causal interpretations and introduce temporal mismatches. Fourth, the GWAS summary statistics used in this study were derived from the Finnish population, predominantly of European ancestry. Such ancestry homogeneity may limit the generalizability of our findings to more genetically diverse populations. Population stratification, although adjusted for using principal components, may still introduce subtle bias. Finally, IVDD phenotyping relied on ICD-10 diagnostic codes, which, although widely used in genetic epidemiology, may lack the granularity needed to distinguish clinical subtypes or disease severity. This potential for misclassification and phenotype heterogeneity could attenuate observed associations or introduce residual bias.

## 6. Conclusions

This study employed a bidirectional two-sample Mendelian randomization (MR) framework based on large-scale genome-wide association study (GWAS) summary data to investigate the potential causal effects between cerebrospinal fluid metabolites (CFMs) and intervertebral disc degeneration (IVDD). Our results provide genetic evidence supporting a causal link, identifying 11 CFMs that may increase susceptibility to IVDD and 6 CFMs with potential protective effects. The reverse MR analysis further demonstrated that IVDD itself can causally influence specific CFMs, with one metabolite positively and 18 metabolites negatively affected, suggesting the possibility of reverse metabolic alterations secondary to disc pathology. By integrating multiple robust MR methodologies, such as inverse variance weighting, the MR-Egger method, and the weighted median method, and by performing extensive sensitivity tests, we minimized the influence of confounding factors, reverse causality, and horizontal pleiotropy. These methodological strengths enhance the reliability of our causal inferences and provide a foundation for future mechanistic exploration. The findings not only expand the current understanding of IVDD pathogenesis from a metabolic perspective but also highlight CFMs as potential biomarkers or targets for early diagnosis and therapeutic intervention. Overall, this study offers novel insights into the metabolic contributions to IVDD. Further research incorporating functional validation, longitudinal metabolic profiling, and systems biology approaches will be essential to clarify the underlying molecular mechanisms and assess clinical applicability.

## Figures and Tables

**Table 1:** MR-Egger method for assessing horizontal pleiotropy of SNPs.

Exposure	Outcome	Egger Intercept	SE	*p* Val
Ornithine levels	IVDD	−0.003	0.007	0.731
Hypoxanthine levels		0.000	0.017	0.979
3-hydroxyhexanoate levels		0.013	0.007	0.076
7-methylxanthine levels		−0.007	0.009	0.414
Beta-alanine levels		−0.002	0.009	0.830
Beta-citrylglutamate levels		−0.006	0.009	0.539
Ethylmalonate levels		0.002	0.005	0.657
Homocarnosine levels		−0.002	0.003	0.495
Isocitrate levels		0.007	0.007	0.296
Mannitol/sorbitol levels		−0.010	0.006	0.109
Methylsuccinate levels		0.006	0.006	0.363
N1-methyl-2-pyridone-5-carboxamide levels		−0.002	0.005	0.683
Palmitoyl dihydrosphingomyelin (d18:0/16:0) levels		0.005	0.006	0.425
X-12104 levels		−0.001	0.008	0.928
X-12906 levels		0.006	0.008	0.454
X-22162 levels		0.003	0.011	0.819
X-24452 levels		−0.002	0.005	0.639

**Table 2:** No significant heterogeneity in IVs of CFMs on IVDD.

Exposure	Outcome	Method	Q	Q_df	Q_*p* Val
Ornithine levels	IVDD	MR-Egger	24.505	16	0.079
Ornithine levels		Inverse variance weighted	24.692	17	0.102
Hypoxanthine levels		MR-Egger	6.170	5	0.290
Hypoxanthine levels		Inverse variance weighted	6.171	6	0.404
3-hydroxyhexanoate levels		MR-Egger	11.638	10	0.310
3-hydroxyhexanoate levels		Inverse variance weighted	16.204	11	0.134
7-methylxanthine levels		MR-Egger	19.801	14	0.137
7-methylxanthine levels		Inverse variance weighted	20.803	15	0.143
Beta-alanine levels		MR-Egger	21.812	12	0.040
Beta-alanine levels		Inverse variance weighted	21.900	13	0.057
Beta-citrylglutamate levels		MR-Egger	15.722	11	0.152
Beta-citrylglutamate levels		Inverse variance weighted	16.297	12	0.178
Ethylmalonate levels		MR-Egger	46.878	44	0.355
Ethylmalonate levels		Inverse variance weighted	47.091	45	0.387
Homocarnosine levels		MR-Egger	59.626	67	0.727
Homocarnosine levels		Inverse variance weighted	60.097	68	0.742
Isocitrate levels		MR-Egger	8.416	17	0.957
Isocitrate levels		Inverse variance weighted	9.579	18	0.945
Mannitol/sorbitol levels		MR-Egger	15.336	18	0.639
Mannitol/sorbitol levels		Inverse variance weighted	18.183	19	0.510
Methylsuccinate levels		MR-Egger	12.798	17	0.750
Methylsuccinate levels		Inverse variance weighted	13.672	18	0.750
N1-methyl-2-pyridone-5-carboxamide levels		MR-Egger	95.730	88	0.269
N1-methyl-2-pyridone-5-carboxamide levels		Inverse variance weighted	95.913	89	0.289
Palmitoyl dihydrosphingomyelin (d18:0/16:0) levels		MR-Egger	33.292	25	0.124
Palmitoyl dihydrosphingomyelin (d18:0/16:0) levels		Inverse variance weighted	34.168	26	0.131
X-12104 levels		MR-Egger	6.234	7	0.513
X-12104 levels		Inverse variance weighted	6.243	8	0.620
X-12906 levels		MR-Egger	6.728	12	0.875
X-12906 levels		Inverse variance weighted	7.327	13	0.885
X-22162 levels		MR-Egger	23.990	16	0.090
X-22162 levels		Inverse variance weighted	24.071	17	0.118
X-24452 levels		MR-Egger	11.820	17	0.811
X-24452 levels		Inverse variance weighted	12.048	18	0.845

**Table 3:** MR-Egger method for assessing horizontal pleiotropy of SNPs in reverse MR analysis.

Exposure	Outcome	Egger Intercept	SE	*p* Val
IVDD	Ornithine levels	−0.001	0.007	0.946
	1-palmitoyl-2-palmitoleoyl-gpc (16:0/16:1) levels	0.010	0.009	0.309
	1-stearoyl-2-oleoyl-gpc (18:0/18:1) levels	0.009	0.010	0.394
	2-aminophenol sulfate levels	0.012	0.031	0.690
	3-(4-hydroxyphenyl)lactate levels	0.009	0.012	0.438
	7-methylxanthine levels	0.031	0.028	0.288
	Diglycerol levels	−0.013	0.012	0.296
	Gluconate levels	−0.016	0.012	0.168
	Glycerophosphoinositol levels	0.003	0.011	0.808
	Guaiacol sulfate levels	0.013	0.026	0.622
	Hippurate levels	0.002	0.024	0.920
	Octanoylcarnitine (c8) levels	0.006	0.031	0.838
	Succinylcarnitine (c4-dc) levels	−0.005	0.011	0.665
	Tryptophan levels	0.005	0.006	0.459
	X-12100 levels	−0.002	0.011	0.835
	Ascorbic acid 3-sulfate levels	0.000	0.023	0.986
	X-23644 levels	0.055	0.054	0.313
	X-24337 levels	0.003	0.011	0.760
	X-24699 levels	−0.004	0.010	0.668

**Table 4:** No significant heterogeneity in IVs of CFMs in reverse MR analysis.

Exposure	**Outcome**	**Method**	**Q**	**Q_df**	**Q_*p* Val**
IVDD	Ornithine levels	MR-Egger	27.226	34	0.788
	Ornithine levels	Inverse variance weighted	27.231	35	0.823
	1-palmitoyl-2-palmitoleoyl-gpc (16:0/16:1) levels	MR-Egger	38.023	34	0.291
	1-palmitoyl-2-palmitoleoyl-gpc (16:0/16:1) levels	Inverse variance weighted	39.216	35	0.286
	1-stearoyl-2-oleoyl-gpc (18:0/18:1) levels	MR-Egger	34.950	34	0.423
	1-stearoyl-2-oleoyl-gpc (18:0/18:1) levels	Inverse variance weighted	35.716	35	0.435
	2-aminophenol sulfate levels	MR-Egger	27.470	34	0.778
	2-aminophenol sulfate levels	Inverse variance weighted	27.632	35	0.808
	3-(4-hydroxyphenyl)lactate levels	MR-Egger	21.051	33	0.947
	3-(4-hydroxyphenyl)lactate levels	Inverse variance weighted	21.667	34	0.950
	7-methylxanthine levels	MR-Egger	28.475	34	0.735
	7-methylxanthine levels	Inverse variance weighted	29.638	35	0.724
	Diglycerol levels	MR-Egger	34.053	34	0.465
	Diglycerol levels	Inverse variance weighted	35.182	35	0.460
	Gluconate levels	MR-Egger	20.209	34	0.971
	Gluconate levels	Inverse variance weighted	22.190	35	0.954
	Glycerophosphoinositol levels	MR-Egger	31.422	34	0.595
	Glycerophosphoinositol levels	Inverse variance weighted	31.481	35	0.639
	Guaiacol sulfate levels	MR-Egger	24.569	34	0.883
	Guaiacol sulfate levels	Inverse variance weighted	24.816	35	0.899
	Hippurate levels	MR-Egger	27.661	34	0.770
	Hippurate levels	Inverse variance weighted	27.671	35	0.806
	Octanoylcarnitine (c8) levels	MR-Egger	29.801	34	0.674
	Octanoylcarnitine (c8) levels	Inverse variance weighted	29.843	35	0.715
	Succinylcarnitine (c4-dc) levels	MR-Egger	43.452	34	0.128
	Succinylcarnitine (c4-dc) levels	Inverse variance weighted	43.696	35	0.149
	Tryptophan levels	MR-Egger	35.026	34	0.419
	Tryptophan levels	Inverse variance weighted	35.603	35	0.440
	X-12100 levels	MR-Egger	24.151	34	0.895
	X-12100 levels	Inverse variance weighted	24.196	35	0.915
	Ascorbic acid 3-sulfate levels	MR-Egger	36.414	34	0.357
	Ascorbic acid 3-sulfate levels	Inverse variance weighted	36.414	35	0.403
	X-23644 levels	MR-Egger	37.286	34	0.320
	X-23644 levels	Inverse variance weighted	38.437	35	0.317
	X-24337 levels	MR-Egger	40.106	34	0.218
	X-24337 levels	Inverse variance weighted	40.218	35	0.250
	X-24699 levels	MR-Egger	33.458	34	0.494
	X-24699 levels	Inverse variance weighted	33.645	35	0.533

## Data Availability

Datasets and supplemental information can be acquired by contacting the corresponding authors.

## Supplementary Materials

The following supporting information can be downloaded at: https://www.mdpi.com/article/10.3390/diagnostics15121526/s1. Supplementary Figure S1 (Figure S1): The scatter plot map presents the causal effect assessment of the relationship between CFM and IVDD risk without SNPs bias estimation with a large effect size. Supplementary Figure S2 (Figure S2): The funnel plot map indicates no significant bias among the individual SNPs’ loci. Supplementary Figure S3 (Figure S3): The leave-one-out sensitivity analysis showed that the association between CFM and IVDD was largely not driven by any individual SNPs. Supplementary Figure S4 (Figure S4): The scatter plot map presents the causal effect assessment of the relationship between IVDD and CFM risk without SNPs bias estimation with a large effect size in reverse MR analysis. Supplementary Figure S5 (Figure S5): The funnel plot map indicates no significant bias among the individual SNPs loci in reverse MR analysis. Supplementary Figure S6 (Figure S6): The leave-one-out sensitivity analysis showed that the association between IVDD and CFM was largely not driven by any individual SNPs in reverse MR analysis.
