# Supplementary figures and images for: Causal Relationship Between Cerebrospinal Fluid Metabolites and Intervertebral Disc Disease: A Bidirectional Mendelian Randomization Study
Source: Diagnostics (Basel). 2025 Jun 16;15(12):1526. doi: 10.3390/diagnostics15121526 (PMC12191516; doi:10.3390/diagnostics15121526)

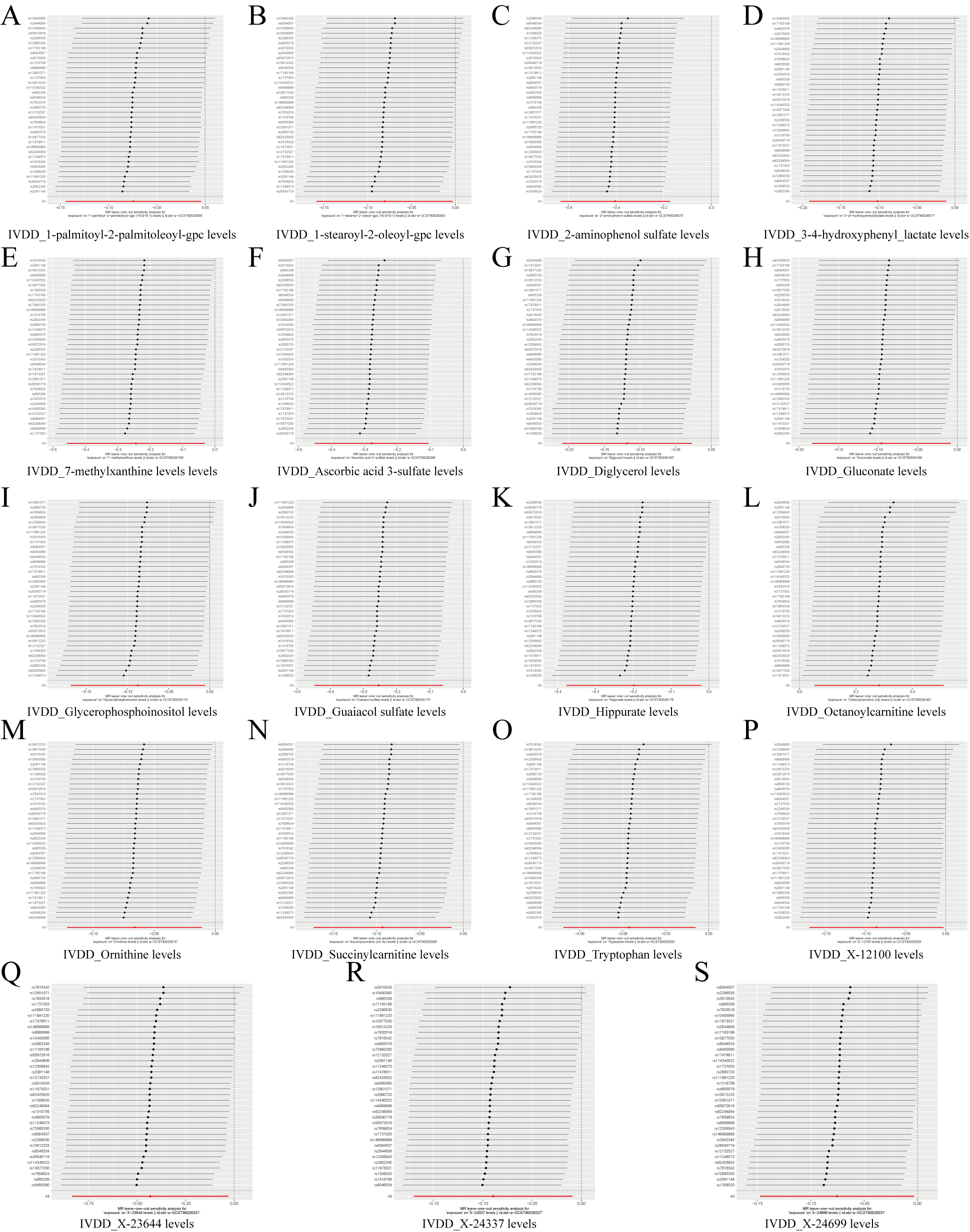

Supplement: Supplementary file 1 [file diagnostics-15-01526-s001.zip › Supplementary Fig. S6.tiff]

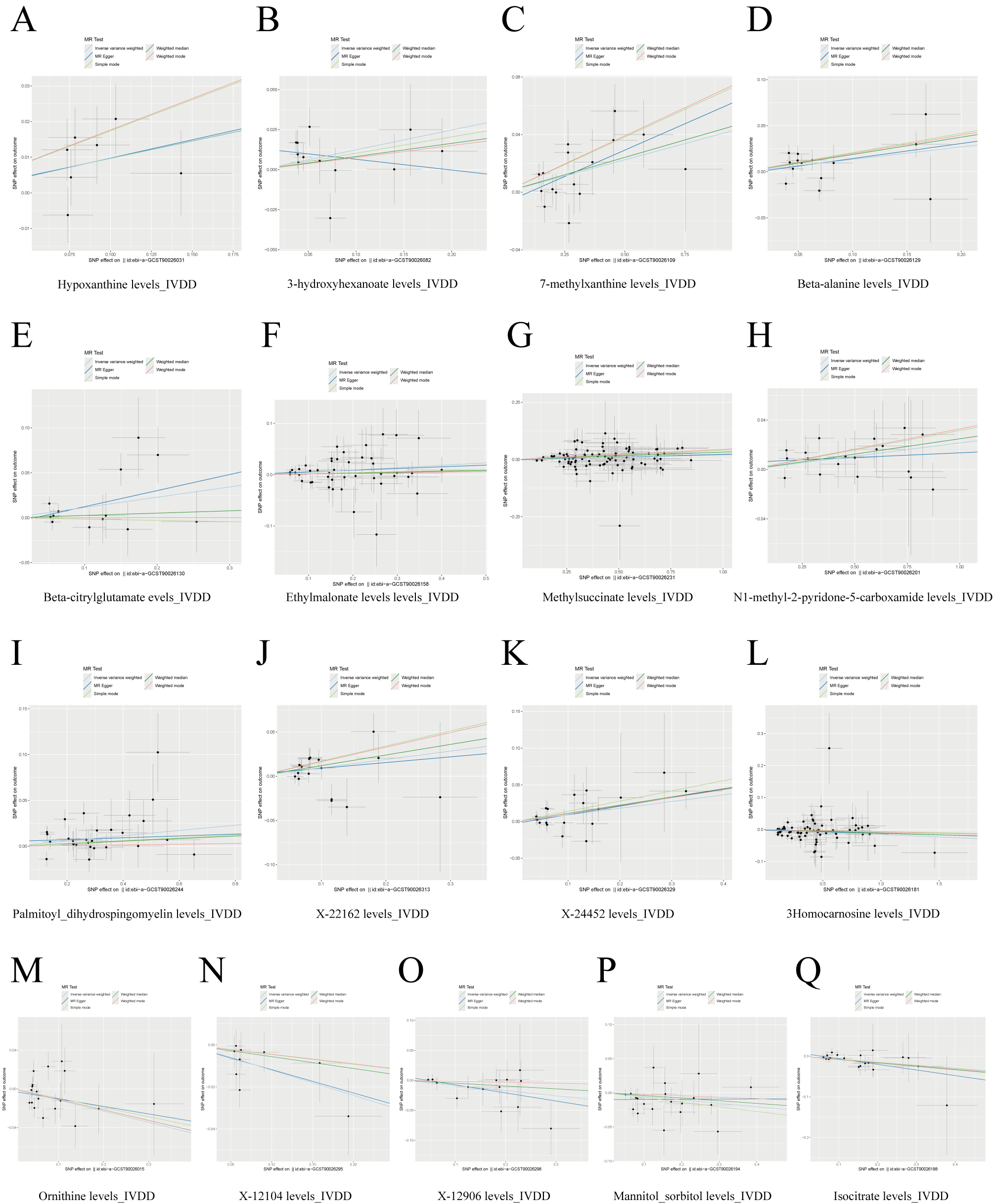

Supplement: Supplementary file 1 [file diagnostics-15-01526-s001.zip › Supplementary Fig. S1.tiff]

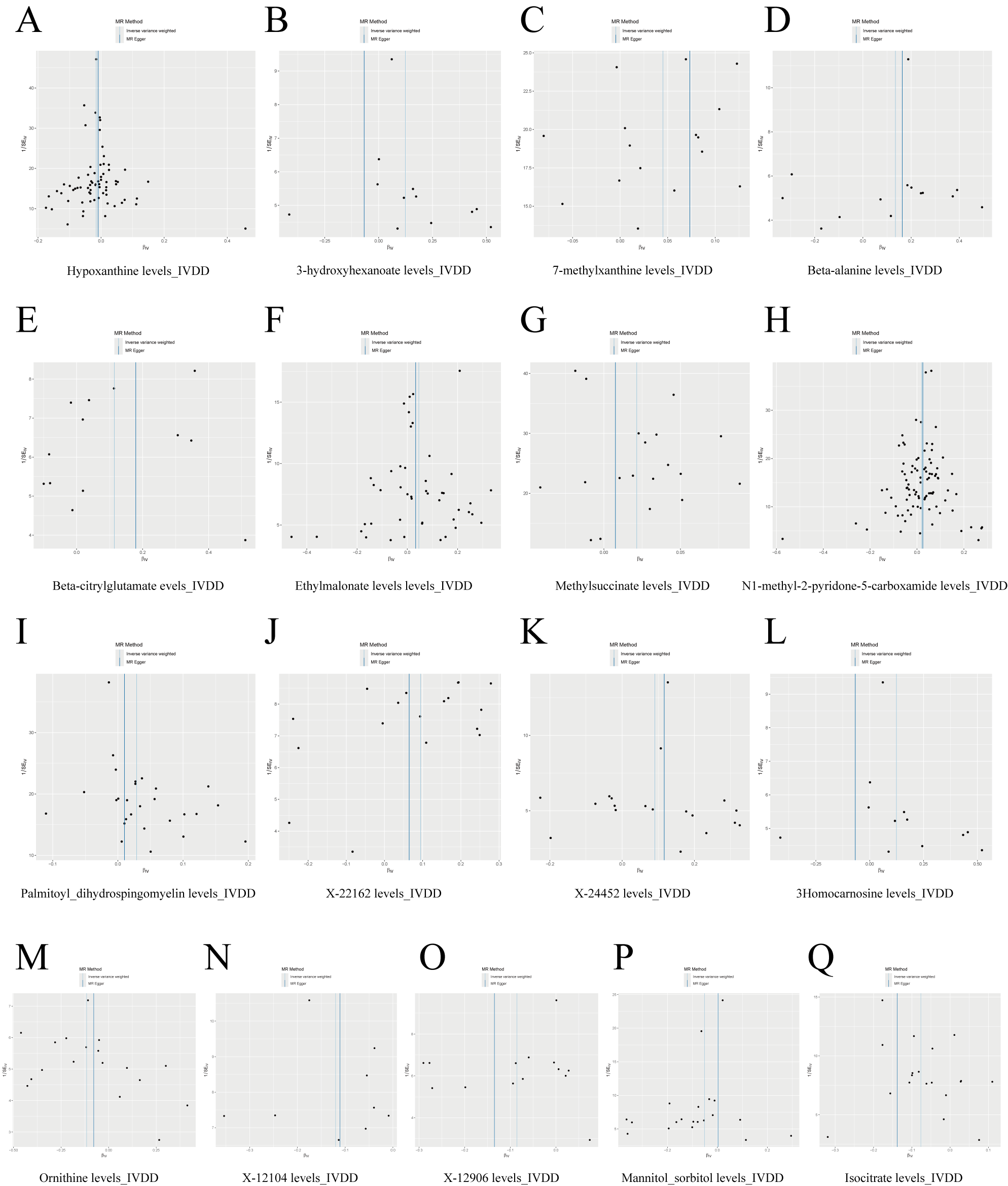

Supplement: Supplementary file 1 [file diagnostics-15-01526-s001.zip › Supplementary Fig. S2.tiff]

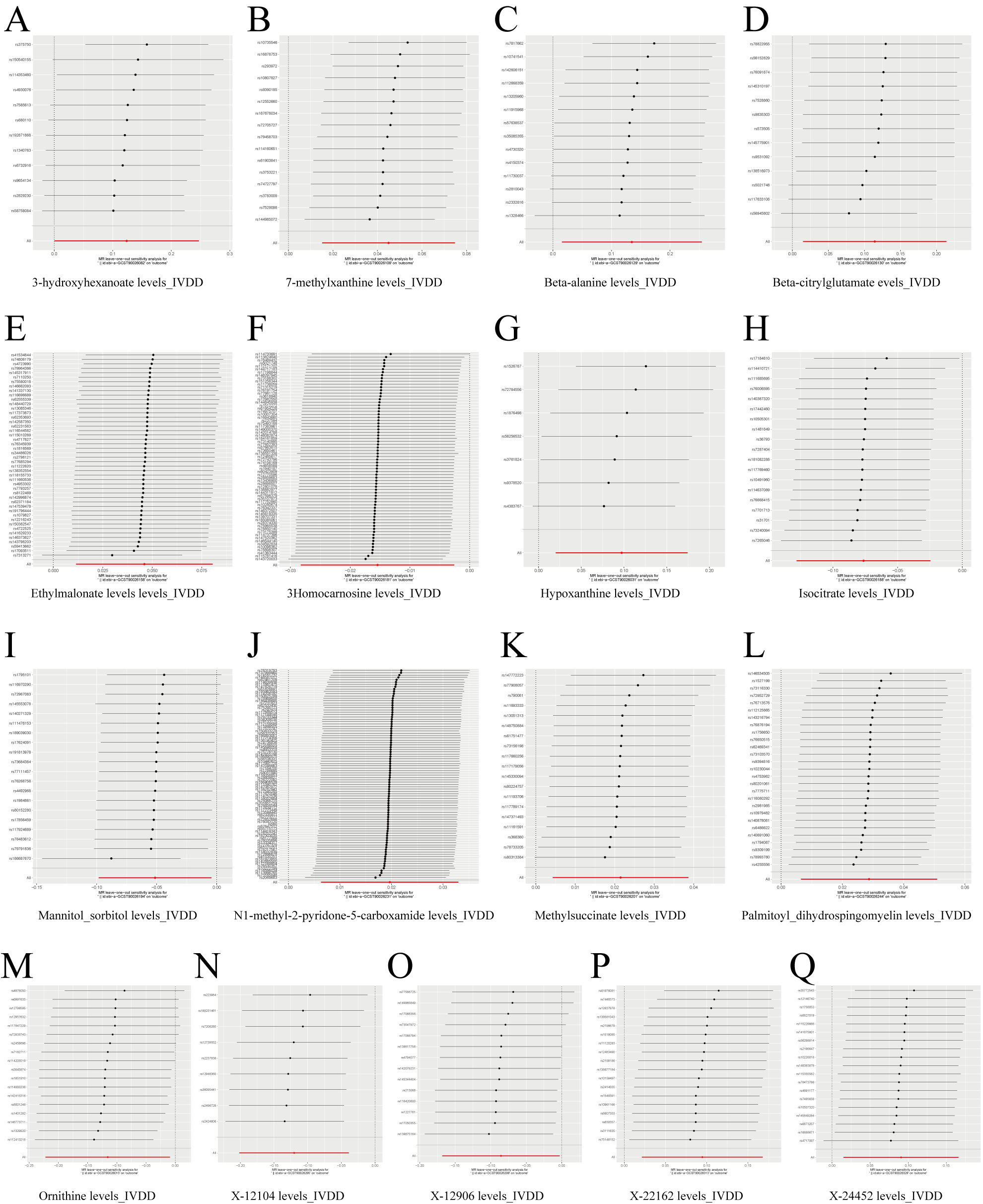

Supplement: Supplementary file 1 [file diagnostics-15-01526-s001.zip › Supplementary Fig. S3.tiff]

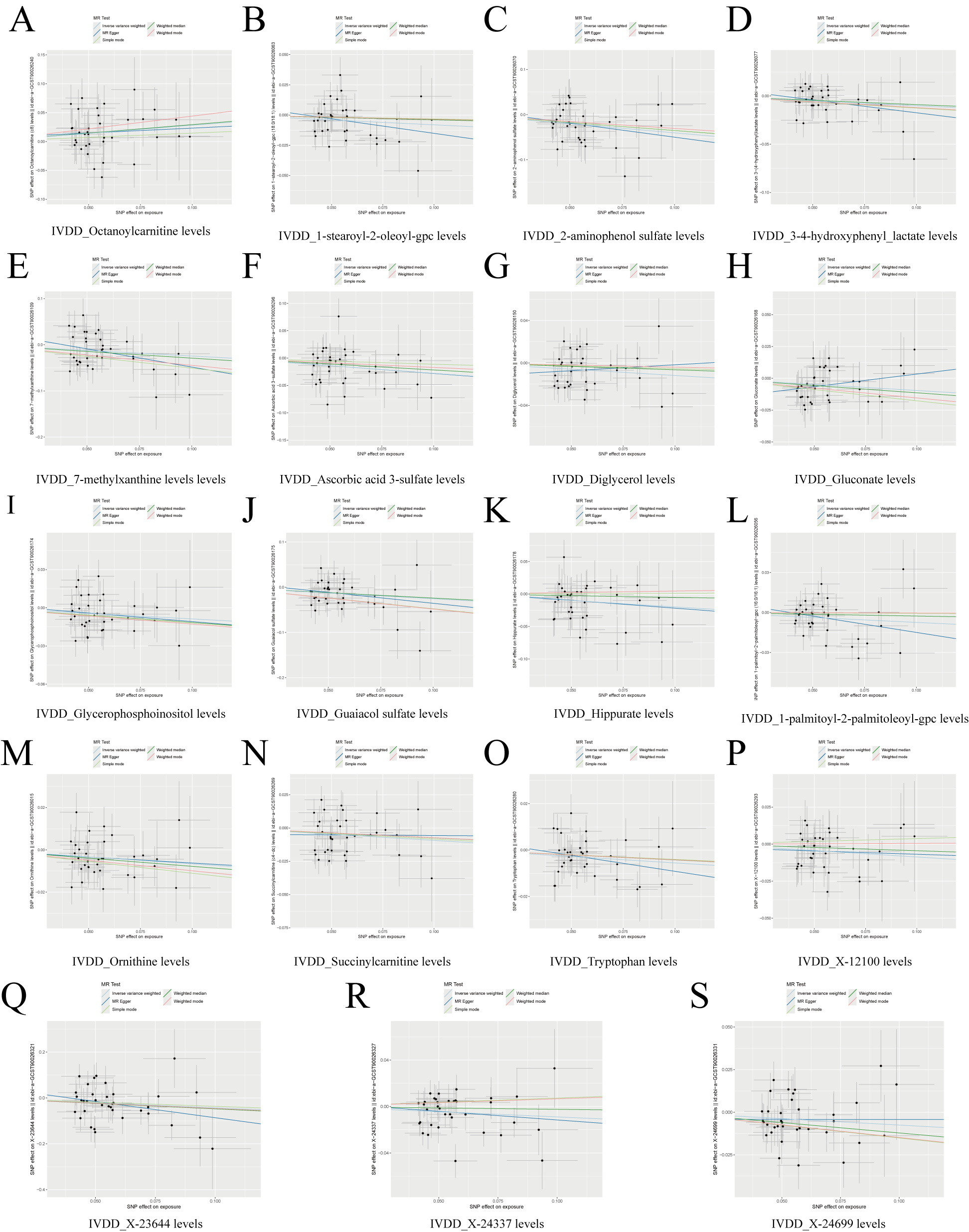

Supplement: Supplementary file 1 [file diagnostics-15-01526-s001.zip › Supplementary Fig. S4.tiff]

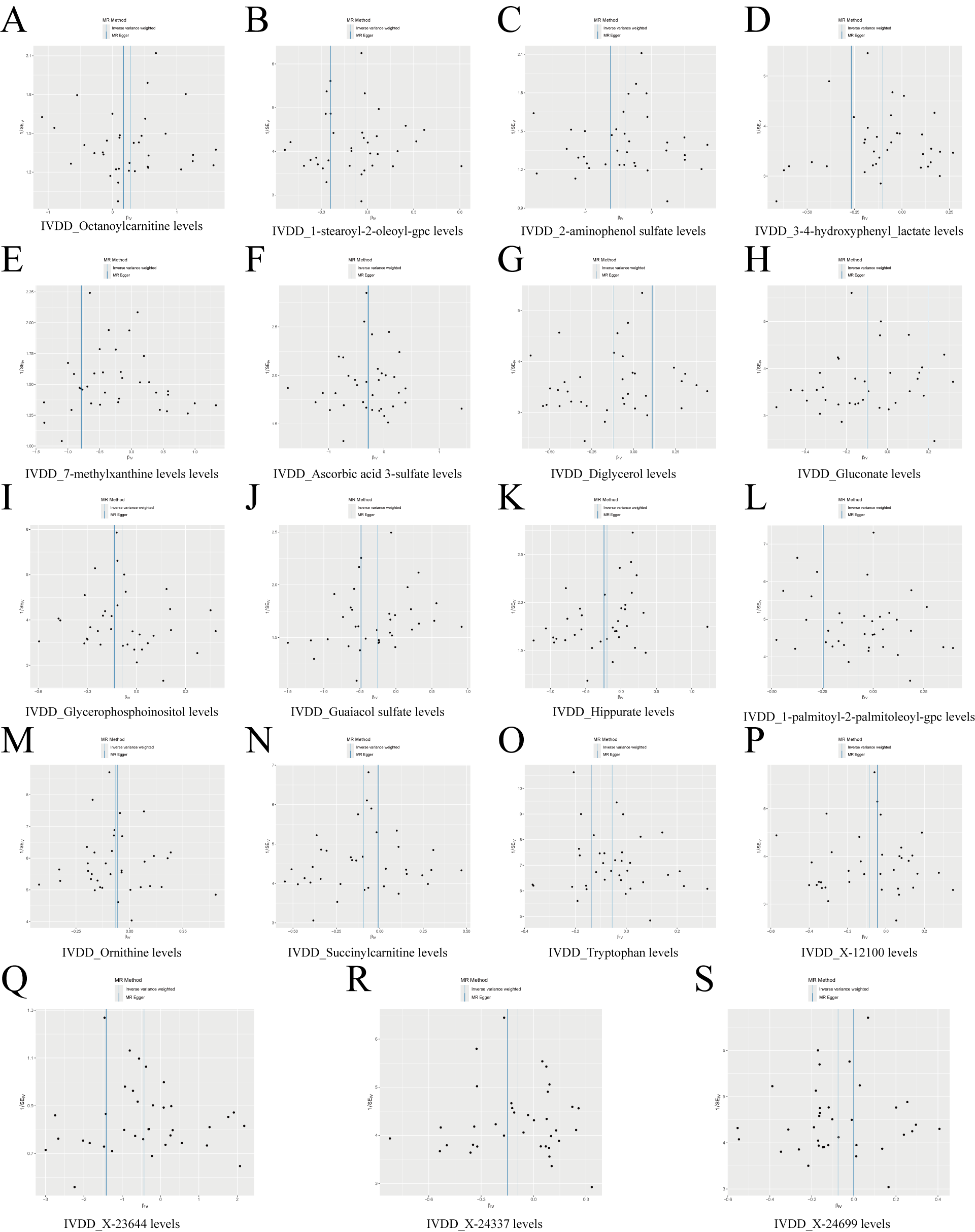

Supplement: Supplementary file 1 [file diagnostics-15-01526-s001.zip › Supplementary Fig. S5.tiff]
